# Machine learning based outcome prediction of microsurgically treated unruptured intracranial aneurysms

**DOI:** 10.1038/s41598-023-50012-8

**Published:** 2023-12-19

**Authors:** Nico Stroh, Harald Stefanits, Alexander Maletzky, Sophie Kaltenleithner, Stefan Thumfart, Michael Giretzlehner, Richard Drexler, Franz L. Ricklefs, Lasse Dührsen, Stefan Aspalter, Philip Rauch, Andreas Gruber, Matthias Gmeiner

**Affiliations:** 1 RISC Software GmbH, Hagenberg, Austria; 2 Department of Neurosurgery, University Medical Centre Hamburg-Eppendorf, Hamburg, Germany; 3 Present Address: Department of Neurosurgery, Kepler University Hospital, Johannes Kepler University, Linz, Austria

**Keywords:** Cerebrovascular disorders, Cerebrovascular disorders

## Abstract

Machine learning (ML) has revolutionized data processing in recent years. This study presents the results of the first prediction models based on a long-term monocentric data registry of patients with microsurgically treated unruptured intracranial aneurysms (UIAs) using a temporal train-test split. Temporal train-test splits allow to simulate prospective validation, and therefore provide more accurate estimations of a model’s predictive quality when applied to future patients. ML models for the prediction of the Glasgow outcome scale, modified Rankin Scale (mRS), and new transient or permanent neurological deficits (output variables) were created from all UIA patients that underwent microsurgery at the Kepler University Hospital Linz (Austria) between 2002 and 2020 (n = 466), based on 18 patient- and 10 aneurysm-specific preoperative parameters (input variables). Train-test splitting was performed with a temporal split for outcome prediction in microsurgical therapy of UIA. Moreover, an external validation was conducted on an independent external data set (n = 256) of the Department of Neurosurgery, University Medical Centre Hamburg-Eppendorf. In total, 722 aneurysms were included in this study. A postoperative mRS > 2 was best predicted by a quadratic discriminant analysis (QDA) estimator in the internal test set, with an area under the receiver operating characteristic curve (ROC-AUC) of 0.87 ± 0.03 and a sensitivity and specificity of 0.83 ± 0.08 and 0.71 ± 0.07, respectively. A Multilayer Perceptron predicted the post- to preoperative mRS difference > 1 with a ROC-AUC of 0.70 ± 0.02 and a sensitivity and specificity of 0.74 ± 0.07 and 0.50 ± 0.04, respectively. The QDA was the best model for predicting a permanent new neurological deficit with a ROC-AUC of 0.71 ± 0.04 and a sensitivity and specificity of 0.65 ± 0.24 and 0.60 ± 0.12, respectively. Furthermore, these models performed significantly better than the classic logistic regression models (*p* < 0.0001). The present results showed good performance in predicting functional and clinical outcomes after microsurgical therapy of UIAs in the internal data set, especially for the main outcome parameters, mRS and permanent neurological deficit. The external validation showed poor discrimination with ROC-AUC values of 0.61, 0.53 and 0.58 respectively for predicting a postoperative mRS > 2, a pre- and postoperative difference in mRS > 1 point and a GOS < 5. Therefore, generalizability of the models could not be demonstrated in the external validation. A SHapley Additive exPlanations (SHAP) analysis revealed that this is due to the most important features being distributed quite differently in the internal and external data sets. The implementation of newly available data and the merging of larger databases to form more broad-based predictive models is imperative in the future.

## Introduction

Unruptured intracranial aneurysms (UIAs) have an estimated prevalence of 3%^1^. Life-threatening intracranial hemorrhages, usually subarachnoid hemorrhage (SAH), are the consequences of UIA rupture with an associated mortality rate of up to 44%^2^.

Owing to the increasing availability and widespread use of neuroradiological imaging, UIAs have been detected more frequently in recent years. The treatment of UIAs aims to minimize or eliminate the risk of rupture. Microsurgical treatment of a UIA should be regarded as a prophylactic intervention, and the indication must be based on an objectifiable benefit-risk assessment. Accordingly, sufficient occlusion of the UIA and the associated elimination of the risk of rupture must prevail over the complication risk of the microsurgical intervention.

However, decision-making in UIAs is complex and many risk factors for aneurysm growth and rupture should be considered to balance the benefits and risks of treatment versus observation. In the case of a high probability of a postoperative complication or a negative outcome, conservative management including clinical and radiological follow-up as well as lifestyle modification or treatment of known risk factors might be more beneficial^3^. Predicting the postoperative outcomes is challenging. There is a large number of potential influencing factors and corresponding data; therefore, the aid of machine learning (ML) algorithms could be helpful in processing and prediction. ML algorithms can analyze large amounts of data and identify complex patterns which might not be achieved by ordinary classifications or logistic regression analysis (LR). A range of ML models have been applied to generate patient-specific predictive analytics for outcomes in neurosurgery, and some studies have demonstrated excellent performance in outcome prediction for a range of neurosurgical conditions^4–6^, particularly cerebrovascular neurosurgery^7–10^.

Several ML-based prediction tools for the complication- and treatment-aware outcomes of patients with aneurysmal subarachnoid hemorrhage (aSAH) have been published^11–14^. However, very few studies have been published on prediction models for UIAs^15,16^.

The aim of this study was to demonstrate that the prediction of early clinical and functional endpoints after microsurgical clipping of UIAs is feasible using advanced ML techniques. As experience and surgical techniques are improving in cerebrovascular centres over time, prediction models need to be continuously adapted. Long-term databases have a clear temporal character, and thus relevant domain shifts must be addressed. This can be accomplished by using temporal train-test splits instead of random splits, to simulate prospective validation on retrospective data. This approach makes it possible to identify those ML algorithms that generalize best from past to future patients. Later, they can be trained on all available data to obtain models for actual clinical use, where a particular focus may even be put on more recent data to account for current and emerging trends in cerebrovascular surgery, and thereby improve the predictive quality of these models. In addition to the prediction model performance on an internal test set, the performance on an independent external data set is of great interest as an external validation of the predictive models.

This study presents the results of the first prediction models based on a long-term monocentric data registry of patients with microsurgically treated UIAs using a temporal train-test split, tested on an internal as well as an external test set.

## Methods

Ethics board approval was obtained prior to data acquisition from the local ethics committee (JKU-Ethikkommission, EC-No.: 1255/2019). All patients or their legal representatives gave their legal informed consent to the surgical procedures and the study conducted in accordance with the Declaration of Helsinki.

Every UIA of the anterior circulation that was microsurgically treated between January 2002 and December 2020 at the Department of Neurosurgery, Kepler University Hospital Linz, was added to the retrospectively collected registry.

The microsurgical operations were all performed using standard approaches and a compilation of the technical intraoperative parameters is shown in Table 1.

**Table 1:** Intraoperative parameters; mRS = modified Rankin Scale, pnND = permanent new neurological deficit, GOS =  Glasgow outcome scale, tnND = transient new neurological deficit, mRS-Diff > 1 = mRS difference > 1 (preoperative vs. postoperative).

Intraoperative parameters	Total	mRS > 2	pnND	GOS < 5	tnND	mRS-Diff > 1	Train Set	Test Set
Number of Aneurysms	466	59	29	63	35	48	380	86
Mean operating duration in minutes (± SD)	250 (± 109)	263 (± 107)	261 (± 108)	291 (± 114)	284 (± 117)	278 (± 115)	226 (± 93)	357 (± 110)
Blood transfusion	4 (0.9%)	2 (3.4%)	0	3 (4.8%)	1 (2.9%)	1 (2.1%)	4 (1.1%)	0
Use of more than 1 clip	163 (35.1%)	24 (40.7%)	11 (37.9%)	25 (40.3%)	12 (35.3%)	20 (42.6%)	134 (35.3%)	29 (34.1%)
Simultaneous clipping of multiple aneurysms	81 (17.4%)	16 (27.1%)	12 (41.4%)	17 (27.0%)	7 (20.0%)	13 (27.1%)	61 (16.1%)	20 (23.3%)
Simultaneous bypass	2 (0.4%)	1 (1.7%)	1 (3.6%)	2 (3.2%)	1 (2.9%)	1 (2.1%)	0	2 (2.4%)
Temporary vessel occlusion	51 (11.1%)	11 (18.6%)	4 (14.3%)	11 (18.0%)	5 (14.3%)	9 (18.8%)	46 (12.2%)	5 (5.9%)
Repositioning of initial clip	88 (19.1%)	14 (23.7%)	8 (28.6%)	17 (27.9%)	9 (25.7%)	10 (20.8%)	83 (22.1%)	5 (5.9%)
Intraoperative rupture	16 (3.5%)	6 (10.2%)	6 (21.4%)	7 (11.3%)	2 (5.7%)	6 (12.5%)	14 (3.7%)	2 (2.4%)
Wrapping	14 (3.0%)	0	0	0	0	0	11 (2.9%)	3 (3.5%)

### Preoperative parameters

Preoperative parameters were divided into patient- and aneurysm-specific parameters and constituted the input variables for the ML algorithms. Patient-specific parameters consisted of basic demographic parameters (age and sex), parameters concerning personal medical history (earlier SAH, hypertension, diabetes mellitus, body mass index (BMI), autosomal dominant polycystic kidney disease, chronic obstructive pulmonary disease, previous stroke, psychiatric disorder, smoking, alcohol abuse, familial frequency of aneurysms), and preoperative scores (PHASES-Score^17^, ASA-Score^18^ (American Society of Anesthesiologists), and modified Rankin Scale (mRS)^19^).

Aneurysm-specific parameters included aneurysm location, calcification, neck diameter, maximum diameter, side, size of the parenteral vessel, morphology, and the occurrence of multiple aneurysms. Preoperative aneurysm-related symptoms such as cranial nerve deficits, epileptic seizures, or aneurysm-related thromboembolic events were also recorded.

### Outcome parameters

Prediction models were calculated for the postoperative parameters. Digital subtraction angiography was performed in every patient to assess complete aneurysm occlusion. New postoperative neurological deficits (nND) were surveyed and divided into transient and permanent nND. A permanent nND persisted after hospital discharge. The functional outcome was assessed using the Glasgow outcome scale (GOS)^20^, mRS^19^, and the difference in the mRS preoperatively to postoperatively. An mRS score of > 2 or a GOS of < 5 was defined as a poor outcome^20,21^. A worsening in mRS of more than one point (postoperatively compared to preoperatively) was regarded as functional deterioration.

### Statistical analysis

Statistical analysis included a univariate descriptive analysis of the collected input and output variables. In addition, an unpenalized LR model was trained on all available features as a simple baseline to quantify the benefit of sophisticated hyperparameter tuning and complex model classes^22^.

### Train-test split

The data were split into training and testing sets. To stimulate prospective validation and obtain reliable estimates of the predictive performance for future patients, we opted for a temporal split, in which the training set consisted of all data until, and including, the year 2018, and the test set consisted of all remaining data from 2019 and 2020. This led to a train-test ratio of 81:19 or 380 vs. 86 samples. Although a single patient can occur multiple times with different aneurysms in the data, ensuring that all corresponding samples appear in either the training or test set was not considered necessary because these samples can safely be assumed to be independent of each other.

### Machine learning algorithms and model selection

A range of ML models was trained on the training set and evaluated on the test set, including extreme gradient boosting estimators (XGB), random forests (RF), extremely randomized trees (ET), support vector machines (SVM), *k*-nearest neighbor classifiers (KNN), generalized additive models (GAM), multilayer perceptrons (MLP), linear discriminant analysis (LDA), and quadratic discriminant analysis (QDA) models. This diverse set of algorithms was selected to make sure we would find the best-performing algorithm for each outcome. Tree-based algorithms, like random forests, are known to work well on tabular data, but including simpler algorithms as well seemed sensible to avoid overfitting due to the small data set.

The hyperparameters of these models were optimized using recent techniques of Bayesian optimization and meta-learning, as implemented in the auto-sklearn package for Python^23^. Hyperparameter optimization not only included finding an optimal model instance but also selecting the optimal preprocessing steps, particularly the class balancing strategy (balancing with respect to class frequencies, vs. no balancing), imputation strategy (mean vs. median imputation for numerical features, most frequent for categorical features), and feature selection. The area under the receiver operating characteristic curve (ROC-AUC) served as the optimization objective because this metric is widely used to illustrate the discriminative power of a binary classifier. Preliminary experiments suggest that optimizing the average precision (AP) does not lead to better overall results. The ROC-AUC was calculated on five predefined train-validation splits of the original training data, where the validation sets were not pairwise disjoint and were biased towards more recent samples from 2017 and 2018, to account for the temporal train-test split. Preliminary experiments suggested that this form of validation was superior to standard *k*-fold cross-validation.

In addition to ROC-AUC and AP, we also reported threshold performance metrics (such as accuracy and sensitivity) on the test set. Analogous to Staartjes et al., the decision thresholds were chosen according to the closest-to-(0, 1) criterion on the training set^15,24^. However, we note that these metrics were only included for the sake of completeness. Because of their strong dependence on a particular decision threshold and the fact that many different threshold selection strategies exist, one must be careful when comparing these metrics between different studies. The ROC-AUC is more robust in this respect and was therefore chosen as the main performance metric.

For estimating the variance of the performance metrics, after fixing hyperparameters, we trained models on 100 bootstrap resamples of the original training set and evaluated them on the test set^25^. The decision threshold was calculated for each of these models individually.

Python version 3.9.7^26^, with scikit-learn 0.24.2^27^, xgboost 1.5.0^28^, pandas 1.4.1^29^, and auto-sklearn 0.14.6^23^ were used for all analyses through the open-source CaTabRa framework^30^. ML models were compared to LR models using the Mann–Whitney *U*-test.

### Feature importance

The SHapely Additive exPlanations (SHAP) framework was used to determine the relevance of individual features to each model and thereby gain insights into the inner workings of otherwise opaque prediction models^31^. In contrast to simpler explanation techniques, such as permutation importance, SHAP also considers interactions between multiple features.

### External validation

We evaluated our models on a retrospectively collected registry from the Department of Neurosurgery of the University Medical Centre Hamburg-Eppendorf, Germany. Apart from new neurological deficits, the registry contained information about the same pre- and postoperative parameters as in our internal data set, and covered the years between 2016 and 2020. A statistical analysis was performed to identify differences in the distribution of the two data sets, focusing on parameters that were deemed important by the SHAP feature importance analysis. The variance of the performance metrics was estimated using the same models that were used for estimating the variance on the internal test set.

## Results

A total of 466 microsurgically treated patients with UIAs were included in the internal data set of this retrospective registry. With a mean age of 55.5 ± 10.5 years, 67.2% of patients were female and 32.8% male. A detailed summary of the 18 preoperative patient-specific parameters is shown in Table 2, and the 10 aneurysm-specific characteristics are listed in Table 3.

**Table 2:** Patient-specific preoperative parameters, with *p*-values for comparing the external set to the internal set; ASA = American Society of Anesthesiologists Classification, ADPKD = autosomal dominant polycystic kidney disease, COPD = chronic obstructive pulmonary disease, DM = Diabetes mellitus, mRS = modified Rankin Scale, SAH = subarachnoid hemorrhage, SD = standard deviation, mRS = modified Rankin Scale, pnND = permanent new neurological deficit, GOS =  Glasgow outcome scale, tnND = transient new neurological deficit, mRS-Diff > 1 = mRS difference > 1 (preoperative vs. postoperative).

Patient-specific preoperative parameters	Internal Set	mRS > 2	pnND	GOS < 5	tnND	mRS-Diff > 1	Train Set	Test Set	External Set	*p*-value
Number of Aneurysms	466	59	29	63	35	48	380	86	256	
Mean Age in years (± SD)	55.5 (± 10.5)	55.9 (± 12.2)	59.8 (± 10.7)	55.9 (± 13.1)	55.2 (± 12.9)	58.1 (± 11.5)	55.1 (± 10.4)	57.3 (± 10.7)	57.4 (± 9.6)	0.0259
Female Gender	313 (67.2%)	31 (52.5%)	22 (75.9%)	34 (54.0%)	23 (65.7%)	30 (62.5%)	257 (67.6%)	56 (65.1%)	198 (77.3%)	0.0041
ASA Classification										< 0.0001
ASA I	101 (21.7%)	3 (5.1%)	3 (10.3%)	6 (9.5%)	10 (28.6%)	5 (10.4%)	93 (24.5%)	8 (9.3%)	2 (0.8%)	
ASA II	255 (54.7%)	28 (57.4%)	15 (51.7%)	30 (47.6%)	15 (42.9%)	27 (56.2%)	200 (52.6%)	55 (64.0%)	157 (61.3%)	
ASA III	101 (21.7%)	24 (40.7%)	10 (34.5%)	23 (36.5%)	8 (22.8%)	14 (29.2%)	80 (21.1%)	21 (24.4%)	95 (37.1%)	
ASA IV	8 (1.7%)	3 (5.1%)	0	3 (4.8%)	2 (5.7%)	1 (2.1%)	6 (1.6%)	2 (2.3%)	2 (0.8%)	
ASA V	1 (0.2%)	1 (1.7%)	1 (3.5%)	1 (1.6%)	0	1 (2.1%)	1 (0.2%)	0	0	
mRS preoperative										< 0.0001
0	288 (61.8%)	14 (23.7%)	15 (51.7%)	19 (30.2%)	15 (42.8%)	29 (60.4%)	222 (58.4%)	66 (76.7%)	124 (48.4%)	
1	107 (23.0%)	14 (23.7%)	8 (27.6%)	17 (27.0%)	13 (37.1%)	14 (29.2%)	89 (23.4%)	18 (20.9%)	102 (39.8%)	
2	51 (10.9%)	12 (20.4%)	4 (13.9%)	13 (20.6%)	5 (14.3%)	4 (8.3%)	51 (13.4%)	0	28 (10.9%)	
3	15 (3.2%)	14 (23.7%)	1 (3.4%)	9 (14.3%)	1 (2.9%)	1 (2.1%)	15 (4.0%)	0	2 (0.8%)	
4	4 (0.9%)	4 (6.8%)	0	4 (6.3%)	1 (2.9%)	0	3 (0.8%)	1 (1.2%)	0	
5	1 (0.2%)	1 (1.7%)	1 (3.4%)	1 (1.6%)	0	0	0	1 (1.2%)	0	
ADPKD	9 (1.9%)	3 (5.1%)	2 (6.9%)	4 (6.3%)	1 (2.9%)	4 (8.3%)	7 (1.9%)	2 (2.5%)	5 (2.0%)	0.9518
Hypertension	282 (60.5%)	38 (64.4%)	19 (65.6%)	37 (58.7%)	22 (62.9%)	32 (66.7%)	225 (59.4%)	57 (67.9%)	159 (62.1%)	0.7516
COPD	70 (15.0%)	12 (20.3%)	6 (20.1%)	11 (17.5%)	4 (11.4%)	9 (18.8%)	63 (16.6%	7 (8.2%)	20 (7.8%)	0.0049
DM II	20 (4.3%)	3 (5.1%)	1 (3.4%)	6 (9.5%)	4 (11.4%)	3 (6.3%)	11 (2.9%)	9 (10.6%)	19 (7.4%)	0.0778
Previous stroke	43 (9.2%)	9 (15.2%)	4 (13.8%)	9 (14.3%)	7 (20.0%)	5 (10.4%)	36 (9.5%)	7 (8.2%)	42 (16.4%)	0.0044
Psychiatric disorder	72 (15.4%)	10 (16.9%)	5 (17.2%)	8 (12.7%)	4 (11.4%)	6 (12.5%)	61 (16.1%)	11 (13.1%)	19 (7.4%)	0.0018
Earlier SAH (another aneurysm)	78 (16.7%)	12 (20.3%)	1 (3.4%)	8 (12.7%)	6 (17.1%)	3 (6.3%)	69 (18.2%)	9 (10.1%)	14 (5.5%)	< 0.0001
Smoking	108 (23.2%)	14 (23.7%)	8 (27.6%)	13 (20.6%)	6 (17.1%)	11 (22.9%)	91 (24.1%)	17 (20.5%)	122 (47.7%)	< 0.0001
Alcohol abuse	30 (6.4%)	6 (10.2%)	2 (6.9%)	6 (9.5%)	1 (2.9%)	3 (6.3%)	25 (6.6%)	5 (6.0%)	21 (8.2%)	0.3936
Aneurysm in family history	22 (4.7%)	3 (5.1%)	2 (6.9%)	3 (4.8%)	2 (5.7%)	2 (4.2%)	20 (5.3%)	2 (2.4%)	22 (8.6%)	0.0390

**Table 3:** Aneurysm-specific preoperative parameters, with *p*-values for comparing the external set to the internal set; MCA = middle cerebral artery, ACA = anterior cerebral artery, AComA = anterior communicating artery, PComA = posterior communicating artery, PCA = posterior cerebral artery, AChA = anterior choroidal artery, mRS = modified Rankin Scale, pnND = permanent new neurological deficit, GOS =  Glasgow outcome scale, tnND = transient new neurological deficit, mRS-Diff > 1 = mRS difference > 1 (preoperative vs. postoperative).

Aneurysm-specific preoperative parameters	Internal Set	mRS > 2	pnND	GOS < 5	tnND	mRS-Diff > 1	Train Set	Test Set	External Set	*p*-value
Number of Aneurysms	466	59	29	63	35	48	380	86	256	
Symptomatic aneurysm	41 (8.8%)	14 (23.7%)	4 (13.8%)	15 (23.8%)	6 (17.1%)	7 (14.6%)	29 (7.6%)	9 (10.5%)	74 (28.9%)	< 0.0001
Calcification	31 (6.7%)	8 (13.6%)	4 (13.8%)	9 (14.3%)	3 (8.6%)	6 (12.5%)	18 (4.7%)	13 (15.1%)	67 (26.2%)	< 0.0001
Aneurysm location										< 0.0001
MCA	309 (66.3%)	33 (55.9%)	12 (41.4%)	34 (54.0%)	23 (65.7%)	21 (43.8%)	255 (67.1%)	54 (62.6%)	164 (64.1%)	
ACA	29 (6.2%)	5 (8.5%)	7 (24.1%)	6 (9.5%)	1 (2.9%)	4 (8.3%)	20 (5.3%)	9 (10.5%)	5 (2%)	
AComA	116 (24.9%)	21 (35.6%)	10 (34.5%)	23 (36.5%)	10 (28.6%)	23 (47.9%)	99 (26.1%)	17 (19.8%)	61 (23.8%)	
PComA	10 (2.1%)	0	0	0	1 (2.9%)	0	5 (1.3%)	5 (5.8%)	16 (6.3%)	
AChA	2 (0.4%)	0	0	0	0	0	1 (0.3%)	1 (1.2%)	10 (3.9%)	
Neck diameter; mean (range) in mm	3.9 (1–12)	4.3 (2–12)	4.1 (2–8)	4.6 (2–12)	4.7 (2–12)	4.3 (2–9)	3.9 (1–12)	3.8 (1–10)	3.0 (1–10)	< 0.0001
Maximum diameter; mean (range) in mm	5.9 (1–25)	7.2 (2–25)	7.0 (3–20)	8.1 (2–25)	7.9 (3–25)	7.7 (3–21)	5.8 (1–25)	6.3 (1–21)	6.0 (1.7–25)	0.1717
Size of parenteral vessel	2.0 (1–3)	2 (1.6–3)	2 (1.7–2.3)	2.0 (1.6–3)	2.1 (1.9–3)	2.0 (2–3)	2.0 (1.1–3)	2.1 (1.7–3)	2.6 (1–6)	< 0.0001
Multiple aneurysms	213 (45.7%)	26 (44.1%)	13 (44.8%)	25 (39.7%)	13 (37.1%)	18 (37.5%)	176 (46.3%)	37 (43.0%)	129 (50.4%)	0.2284
Irregular morphology / Lobulation	172 (36.9%)	30 (50.8%)	17 (58.6%)	32 (50.8%)	12 (34.3%)	27 (56.3%)	145 (38.4)	27 (31.4%)	71 (27.7%)	0.0101

Intraoperative parameters were collected as listed in Table 1. For the establishment of the preoperative prediction models, these parameters were not used, with the exception of “simultaneous clipping of multiple aneurysms”, because this parameter is actually already preoperatively known and therefore applicable for a preoperative prediction model.

Postoperatively, 35 patients (7.5%) presented with a transient nND, and 29 (6.2%) had a permanent nND. A good functional outcome, corresponding to a GOS of ≥ 5, was identified in 403 patients (86.5%). The postoperative mRS was < 2 in 407 patients (87.3%), whereas after subtracting the preoperative baseline mRS, only 48 patients (10.3%) had a worsening in mRS of > 1, in the sense of an objectifiable functional deterioration. All the outcome parameters are listed in Table 4.

**Table 4:** Outcome Parameters, with *p*-values for comparing the external set to the internal set; GOS =  Glasgow outcome scale, mRS = modified Rankin Scale, preop = preoperative, postop = postoperative; *no prediction models were made for this outcome parameter.

Outcome parameters	Internal Set	Train Set	Test Set	External Set	*p*-value
New neurological deficit	64 (13.7%)	52 (13.7%)	12 (14.0%)		
Transient	35 (7.4%)	29 (7.6%)	6 (7.0%)		
Permanent	29 (6.3%)	23 (6.1%)	6 (7.0%)		
mRS > 2	59 (12.8%)	51 (13.4%)	8 (9.3%)	11 (4.3%)	< 0.0001
mRS difference > 1 (preop vs. postop.)	48 (10.2%)	40 (10.5%)	8 (9.3%)	9 (3.5%)	0.0004
GOS < 5	63 (13.6%)	51 (13.4%)	12 (14.0%)	19 (7.4%)	0.0043
Complete angiographical occlusion*	459 (98.5%)	373 (98.2%)	86 (100.0%)	248 (96.9%)	0.1445

**Figure 1:**
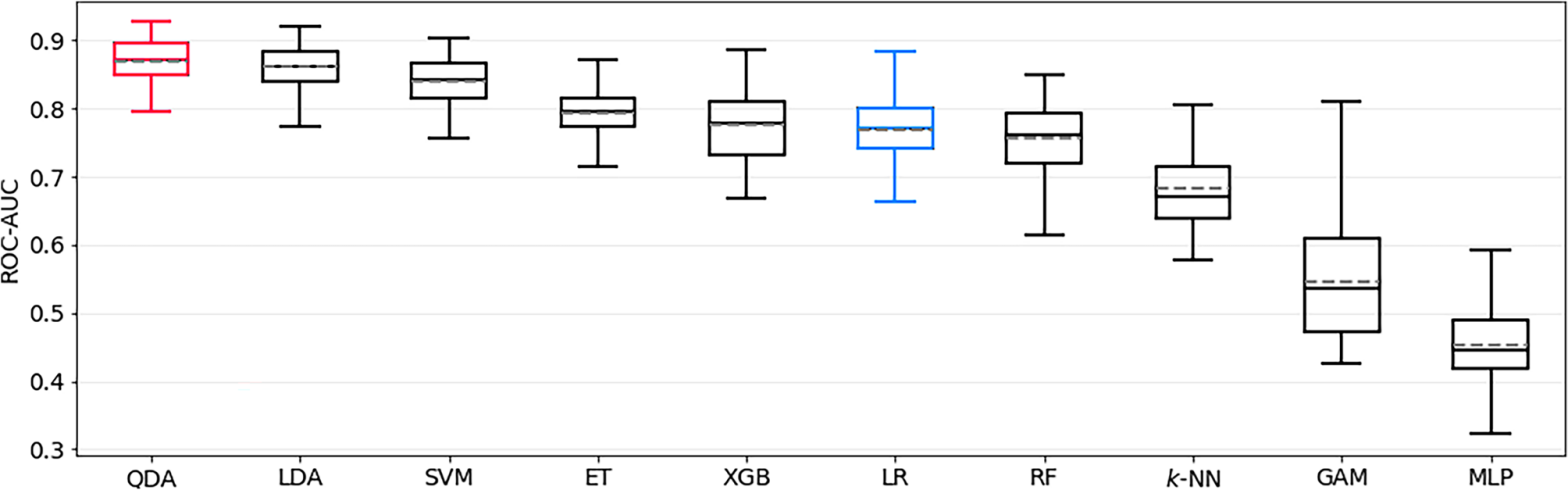
Bootstrapped test-set ROC-AUC of all models trained to predict postoperative mRS > 2, sorted by mean ROC-AUC. QDA is the top-performing model, and LR represents the logistic regression baseline model (both highlighted). mRS = modified Rankin Scale, ROC-AUC = area under Receiver Operating Characteristic curve, QDA = quadratic discriminant analysis, ET = Extremely Randomized Trees, SVM = support vector machine, LDA = linear discriminant analysis, XGB = extreme gradient boosting, RF = Random Forest, KNN = *k*-nearest neighbors, GAM = generalized additive model, MLP = Multilayer Perceptron.

**Figure 2:**
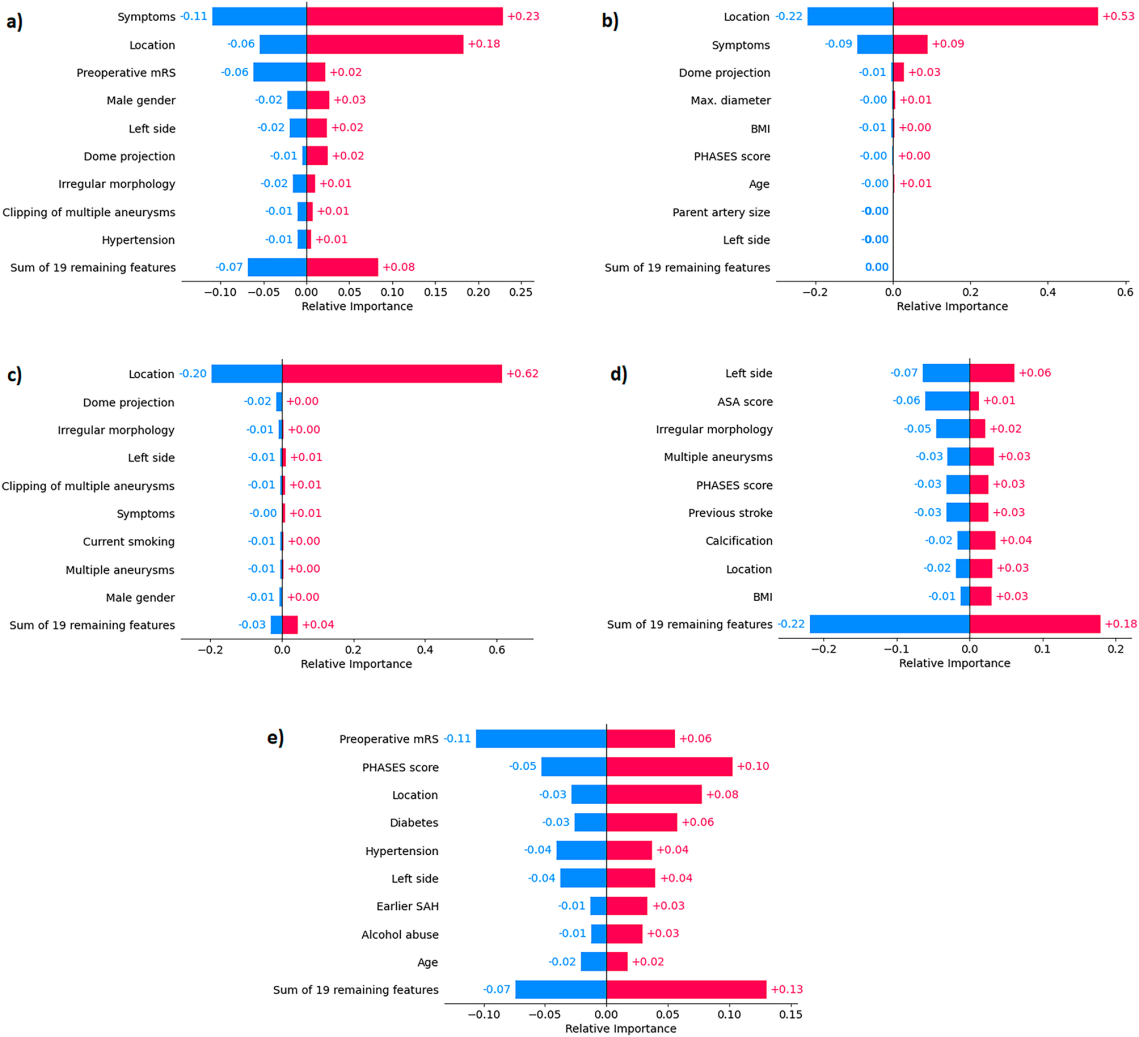
SHAP feature importance of the best prediction models for each task (**a**–**e**). For every feature, negative and positive average contributions are depicted separately, in bluish and reddish hues, respectively. (**a**) mRS > 2, (**b**) mRS-difference > 1, (**c**) permanent nND, (**d**) transient nND, (**e**) GOS < 5. mRS = modified Rankin Scale, BMI = body mass index, nND = new neurological deficit, ADPKD = autosomal dominant polycystic kidney disease, GOS =  Glasgow outcome scale.

The best model for predicting postoperative mRS > 2 was a QDA estimator, which achieved a ROC-AUC of 0.87 ± 0.03. This model significantly outperformed the LR baseline, which achieved only 0.77 ± 0.05 (*p* < 0.0001). The ROC-AUC of all models trained to predict this outcome is shown in Fig. 1. The sensitivity and specificity of the QDA model were 0.83 ± 0.08 and 0.71 ± 0.07, respectively. SHAP identified preoperative aneurysm-related symptoms, aneurysm location, and preoperative mRS as the most important features; see Fig. 2a for details.

**Figure 3:**
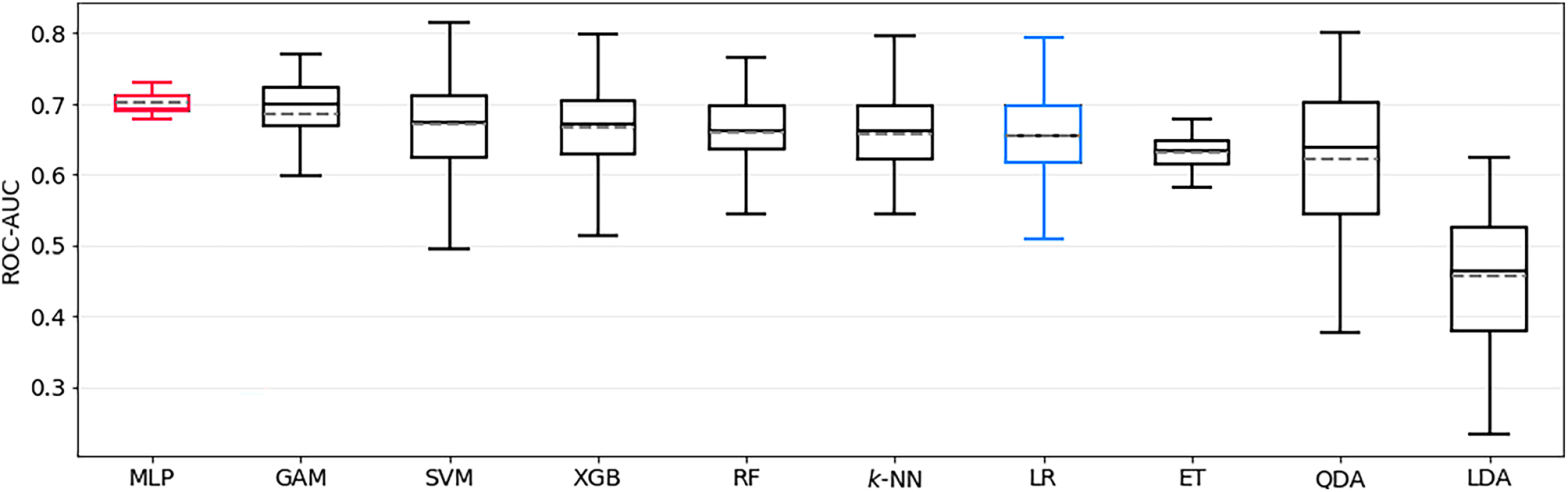
Bootstrapped test-set ROC-AUC of all models trained to predict postoperative mRS-difference > 1, sorted by mean ROC-AUC. MLP is the top-performing model, and LR represents the logistic regression baseline model (both highlighted). mRS = modified Rankin Scale, ROC-AUC = area under Receiver Operating Characteristic curve, MLP = multilayer perceptron, GAM = generalized additive model, SVM = support vector machine, XGB = extreme gradient boosting, RF = Random Forest, KNN = k-nearest neighbors, LR = logistic regression, ET = Extremely Randomized Trees, QDA = quadratic discriminant analysis, LDA = linear discriminant analysis.

The best model for predicting post- to preoperative mRS difference > 1 was a MLP, with a ROC-AUC of 0.70 ± 0.02 in the test set. The LR baseline, which achieved 0.65 ± 0.06, was significantly outperformed (*p* < 0.0001) by the MLP model. The ROC-AUC of all models trained to predict this outcome is shown in Fig. 3. The sensitivity and specificity of the MLP were 0.74 ± 0.07 and 0.50 ± 0.04, respectively. SHAP identified aneurysm location, preoperative aneurysm-related symptoms and dome projection as the most important features; see Fig. 2b for details.

**Figure 4:**
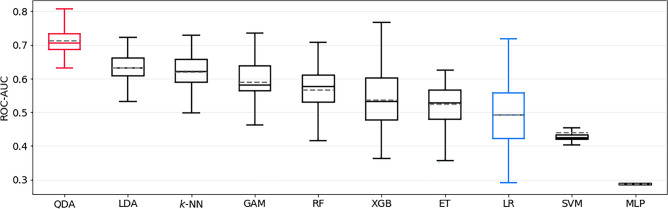
Bootstrapped test-set ROC-AUC of all models trained to predict permanent new neurological deficit (pnND), sorted by mean ROC-AUC. QDA is the top-performing model, and LR represents the logistic regression baseline model (both highlighted). ROC-AUC = area under Receiver Operating Characteristic curve, QDA = quadratic discriminant analysis, LDA = linear discriminant analysis, KNN = k-nearest neighbors, GAM = generalized additive model, RF = Random Forest, XGB = extreme gradient boosting, ET = Extremely Randomized Trees, LR = logistic regression, SVM = support vector machine, MLP = multilayer perceptron.

The best model for predicting permanent nND was QDA, achieving a ROC-AUC of 0.71 ± 0.04 on the test set and significantly outperforming the LR baseline with 0.49 ± 0.09 (*p* < 0.0001). The ROC-AUC of all models trained to predict this outcome is shown in Fig. 4. Sensitivity and specificity were 0.65 ± 0.24 and 0.60 ± 0.12, respectively. Aneurysm location was identified as the single most important feature, as shown in Fig. 2c.

**Figure 5:**
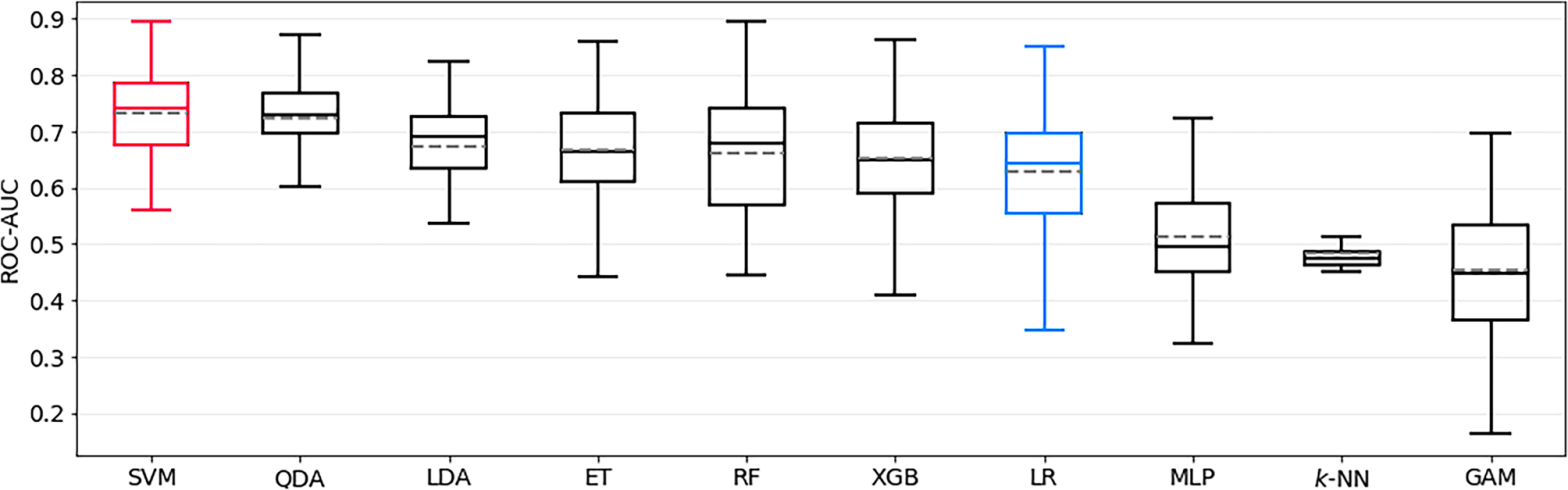
Bootstrapped test-set ROC-AUC of all models trained to predict transient new neurological deficit (tnND), sorted by mean ROC-AUC. SVM is the top-performing model, and LR represents the logistic regression baseline model (both highlighted). ROC-AUC = area under Receiver Operating Characteristic curve, SVM = support vector machine, QDA = quadratic discriminant analysis, LDA = linear discriminant analysis, ET = Extremely Randomized Trees, RF = Random Forest, XGB = extreme gradient boosting, LR = logistic regression, MLP = multilayer perceptron, KNN = k-nearest neighbors, GAM = generalized additive model.

The best model for predicting transient nND was a SVM estimator, achieving a ROC-AUC of 0.73 ± 0.07 on the test set. The LR baseline performed again significantly worse, with 0.63 ± 0.11 (*p* < 0.0001). The ROC-AUC of all models trained to predict this outcome is shown in Fig. 5. The sensitivity and specificity of the SVM model were 0.00 ± 0.02 and 0.97 ± 0.03, respectively, indicating a non-optimal threshold selection strategy in this case. The side of the aneurysm, ASA score and aneurysm morphology (regular vs. irregular) were identified as the most important features in this model (Fig. 2d).

**Figure 6:**
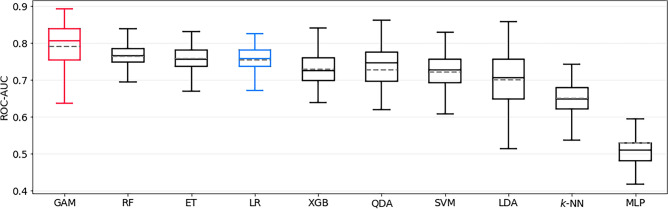
Bootstrapped test-set ROC-AUC of all models trained to predict GOS < 5, sorted by mean ROC-AUC. GAM is the top-performing model, and LR represents the logistic regression baseline model (both highlighted). GOS =  Glasgow outcome scale, ROC-AUC = area under Receiver Operating Characteristic curve, GAM = generalized additive model, RF = Random Forest, ET = Extremely Randomized Trees, LR = logistic regression, XGB = extreme gradient boosting, QDA = quadratic discriminant analysis, SVM = support vector machine, LDA = linear discriminant analysis, KNN = k-nearest neighbors, MLP = multilayer perceptron.

The best model for predicting GOS < 5 was the GAM estimator, achieving a ROC-AUC of 0.79 ± 0.07 on the test set. The LR baseline performed significantly worse, with 0.75 ± 0.04 (*p* < 0.0001). The ROC-AUC of all models trained to predict this outcome is shown in Fig. 6. The sensitivity and specificity of the GAM were 0.69 ± 0.12 and 0.73 ± 0.06, respectively. Preoperative mRS score, PHASES score, and aneurysm location were identified as the most important features in this model, as shown in Fig. 2e.

All the performance metrics are summarized in Table 5.

**Table 5:** Test-set performance of the best model and baseline logistic regression model for each outcome, displayed as mean ± std.dev. Statistically significant differences between best- and baseline models in terms of ROC-AUC and Average Precision are marked as * (Mann–Whitney U test, alpha = 0.05).

Outcome	Model	ROC-AUC	*p*-value	Average Prec	*p*-value	Accuracy	Sensitivity	Specificity	PPV	NPV
mRS > 2	QDA	0.87 ± 0.03*	*p* < 0.0001	0.60 ± 0.13*	*p* < 0.0001	0.72 ± 0.06	0.83 ± 0.08	0.71 ± 0.07	0.24 ± 0.04	0.98 ± 0.01
	Baseline LR	0.77 ± 0.05		0.40 ± 0.08		0.79 ± 0.05	0.51 ± 0.14	0.82 ± 0.06	0.24 ± 0.07	0.94 ± 0.01
mRS-Diff. > 1	MLP	0.70 ± 0.02*	*p* < 0.0001	0.19 ± 0.05	*p* = 0.2561	0.52 ± 0.03	0.74 ± 0.07	0.50 ± 0.04	0.13 ± 0.00	0.95 ± 0.01
	Baseline LR	0.65 ± 0.06		0.19 ± 0.06		0.66 ± 0.07	0.50 ± 0.16	0.67 ± 0.08	0.14 ± 0.03	0.93 ± 0.02
perm. nND	QDA	0.71 ± 0.04*	*p* < 0.0001	0.26 ± 0.08*	*p* < 0.0001	0.60 ± 0.10	0.65 ± 0.24	0.60 ± 0.12	0.11 ± 0.02	0.96 ± 0.02
	Baseline LR	0.49 ± 0.09		0.08 ± 0.02		0.69 ± 0.07	0.19 ± 0.16	0.73 ± 0.08	0.05 ± 0.04	0.92 ± 0.01
trans. nND	SVM	0.73 ± 0.07*	*p* < 0.0001	0.15 ± 0.05*	*p* = 0.0116	0.90 ± 0.03	0.00 ± 0.02	0.97 ± 0.03	0.22 ± 0.41	0.93 ± 0.00
	Baseline LR	0.63 ± 0.11		0.19 ± 0.10		0.74 ± 0.05	0.41 ± 0.19	0.77 ± 0.08	0.12 ± 0.06	0.95 ± 0.02
GOS < 5	GAM	0.79 ± 0.08*	*p* < 0.0001	0.45 ± 0.09	*p* = 0.0879	0.73 ± 0.05	0.69 ± 0.12	0.73 ± 0.06	0.30 ± 0.05	0.93 ± 0.02
	Baseline LR	0.75 ± 0.04		0.43 ± 0.09		0.74 ± 0.05	0.57 ± 0.13	0.77 ± 0.06	0.30 ± 0.06	0.92 ± 0.02

The QDA and GAM models for mRS > 2, permanent nND and GOS < 5 perform best in terms of Average Precision, too. mRS = modified Rankin Scale, GOS =  Glasgow outcome scale, nND = new neurological deficit, LR = logistic regression, QDA = quadratic discriminant analysis, MLP = multilayer perceptron, SVM = support vector machine, GAM = generalized additive model, ROC-AUC = area under receiver operating characteristic curve, PPV = positive predictive value, NPV = negative predictive value.

The external validation set contained 256 patients with a mean age of 57.4 ± 9.6 years. 77.3% of the patients were female and 22.7% male. A detailed summary of the preoperative patient-specific parameters is shown in Table 2, and the aneurysm-specific characteristics are listed in Table 3. Most of the preoperative parameters differ significantly from the internal data set. In particular, this applies to all parameters that were found most relevant by the SHAP feature importance analysis, namely aneurysm-related symptoms, aneurysm location and preoperative mRS (*p* < 0.0001).

A good functional outcome, corresponding to a GOS of ≥ 5, was identified in 237 patients (92.6%). The postoperative mRS was ≤ 2 in 245 patients (95.7%), whereas after subtracting the preoperative baseline mRS, only 9 patients (3.5%) had a worsening in mRS of > 1, in the sense of an objectifiable functional deterioration. All the outcome parameters are listed in Table 4. New neurological deficits were not recorded in the external validation set. Similar to the preoperative parameters, the postoperative outcomes also differ significantly from the internal set.

The QDA estimator that best predicted postoperative mRS > 2 on our internal test set only achieved a ROC-AUC of 0.61 ± 0.03 in external validation. The LR baseline generalized slightly better to the external set, with a ROC-AUC of 0.66 ± 0.04.

The MLP estimator that best predicted post- to preoperative mRS difference > 1 on our internal test set achieved a ROC-AUC of 0.53 ± 0.01 in external validation. The LR baseline showed equally poor discrimination (0.53 ± 0.03).

The GAM model that best predicted GOS < 5 on our internal test set achieved a ROC-AUC of 0.58 ± 0.03 in external validation. It was outperformed by the LR baseline, with 0.62 ± 0.02.

**Figure 7:**
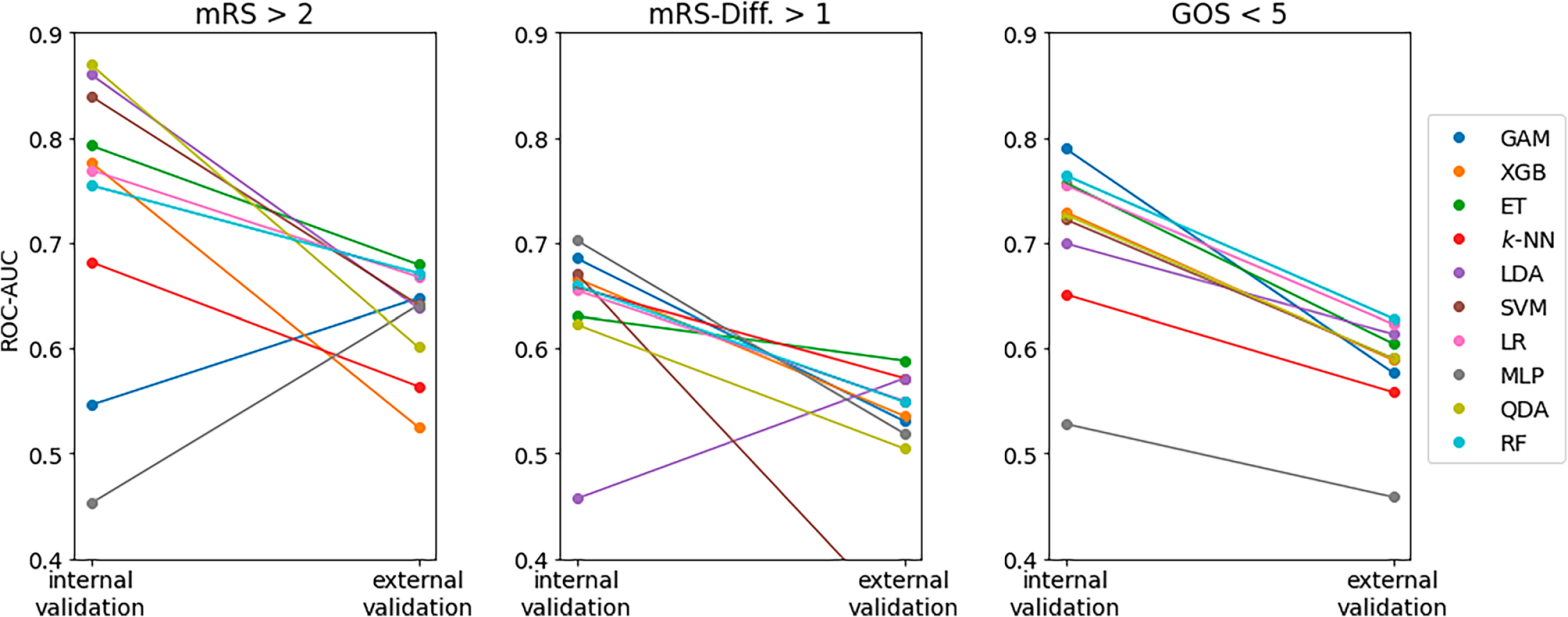
ROC-AUC of all models on both the internal (left column in each subplot) and external (right column in each subplot) test set. One can clearly observe the pronounced performance drop, especially of the model with the highest ROC-AUC on the internal test set. ROC-AUC = area under Receiver Operating Characteristic curve, mRS = modified Rankin Scale, GOS =  Glasgow outcome scale, GAM = Generalized Additive Model, XGB = extreme gradient boosting, ET = Extremely Randomized Trees, k-NN = k-nearest neighbors, LDA = linear discriminant analysis, SVM = support vector machine, LR = logistic regression, MLP = Multilayer Perceptron, QDA = quadratic discriminant analysis, RF = Random Forest.

All the performance metrics of external validation are summarized in Table 6. The performance drop of the respective best model and the LR baseline compared to the internal test set is always significant, for each outcome (*p* < 0.0001). Figure 7 additionally depicts the ROC-AUC of all trained models on both the internal test set and the external set, illustrating that the best models on the internal test set are always outperformed by other models on the external set. Extra Trees and Random Forests seem to generalize best to the external validation set.

**Table 6:** External validation performance of the best model (on the internal test set) and baseline logistic model for each outcome, displayed as mean ± std.dev. Note that transient nND was not recorded in the external data, so no results are available for that outcome.

Outcome	Model	ROC-AUC	Average Prec	Accuracy	Sensitivity	Specificity	PPV	NPV
mRS > 2	QDA	0.61 ± 0.03	0.08 ± 0.01	0.57 ± 0.04	0.59 ± 0.06	0.57 ± 0.05	0.06 ± 0.01	0.97 ± 0.01
	Baseline LR	0.66 ± 0.04	0.16 ± 0.04	0.69 ± 0.07	0.55 ± 0.10	0.69 ± 0.07	0.08 ± 0.01	0.97 ± 0.01
mRS-Diff. > 1	MLP	0.53 ± 0.01	0.05 ± 0.01	0.54 ± 0.05	0.44 ± 0.04	0.54 ± 0.05	0.03 ± 0.01	0.96 ± 0.00
	Baseline LR	0.53 ± 0.03	0.11 ± 0.05	0.64 ± 0.09	0.48 ± 0.12	0.65 ± 0.09	0.05 ± 0.01	0.97 ± 0.00
GOS < 5	GAM	0.58 ± 0.03	0.12 ± 0.02	0.59 ± 0.07	0.49 ± 0.13	0.60 ± 0.09	0.09 ± 0.01	0.94 ± 0.01
	Baseline LR	0.62 ± 0.02	0.16 ± 0.03	0.67 ± 0.05	0.47 ± 0.09	0.68 ± 0.06	0.11 ± 0.01	0.94 ± 0.01

mRS = modified Rankin Scale, GOS =  Glasgow outcome scale, nND = new neurological deficit, LR = logistic regression, QDA = quadratic discriminant analysis, MLP = multilayer perceptron, SVM = support vector machine, GAM = generalized additive model, ROC-AUC = area under receiver operating characteristic curve, PPV = positive predictive value, NPV = negative predictive value.

## Discussion

In recent years, ML-based predictive models have become increasingly important in medical sciences, including neurosurgery. To date, numerous well-performing prediction models have been published, e.g. for neurooncology^32^, spinal research^4,5^, and cerebrovascular pathologies. Aneurysm detection using computer-aided diagnosis systems is one example^33–36^. Such models should be regarded as a support or supplement and not as a substitute for the clinical decision process^37,38^. ML has further applications in distinguishing rupture status or rupture risk assessment^9,39,40^. In the study by Zhu et al., ML-based models were shown to be superior to previously established prediction scores (e.g., PHASES score) as well as classic LR analysis^41^.

Regarding outcome prediction, several ML models have already been published that focus on functional outcomes after aSAH^7,11,13,14,42,43^. Muscas et al. and Ramos et al. developed relevant models for complication prediction, especially shunt-dependent hydrocephalus and delayed cerebral ischemia, respectively^12,44^. Thus far, prediction models for post-treatment occlusion rates are only available for endovascular-treated aneurysms^45–47^. Postoperative occlusion rates in microsurgically treated aneurysms are traditionally very high^48^. In this series, 98.5% of all treated aneurysms and 100% of those in the test set were completely occluded. Therefore, no prediction models were trained and evaluated for this outcome. Decision-making in diagnosed UIA is complex and always requires balancing the risk of rupture with that of preventive treatment. Strategies to improve risk stratification and outcome prediction remain rare and are therefore highly warranted. Staartjes et al. addressed this issue in their pilot study and were able to demonstrate the feasibility of such predictive models for functional outcomes and postoperative complications^15^. Moreover, Ishankulov et al. published promising predictive models for a functional outcome (mRS) after the treatment of UIAs in a pilot study^16^. However, both studies randomly assigned their patients to either the train or test group (random train-test split)^49^.

Owing to the continuous improvement in surgical standards in recent years, we believe that training sets have a clear temporal character, and thus relevant domain shifts must be addressed. Therefore, to guarantee realistic assessments of our prediction models in a clinical setting, we opted to employ a temporal train-test split. Temporal splits allow the approximation of the predictive quality of a model when applied to future patients more accurately than random splits^50^, and therefore are the natural candidate for simulating prospective validation in retrospective studies. They do have several drawbacks, though, like producing models with limited generalizability, which necessitates re-training the models on all available data before an actual prospective validation or deployment to clinical practice takes place. Analogous to our modified cross-validation strategy, it may then even be beneficial to pay more attention to more recent samples for further maximizing the generalizability to future data. The temporal validation strategy presented in this work merely seeks to provide honest estimates of what can be expected from a prospective validation. Irrespective of that, any prediction model currently used in clinical practice should be continuously re-evaluated and re-trained when new data become available to account for possible negative effects of domain shifts.

Our models showed an excellent or at least acceptable discrimination performance for the most important outcome parameters, such as permanent nND, postoperative mRS, and mRS difference. Currently, ROC-AUC is regarded as a reliable parameter for comparing different ML models^51,52^.

In our study, the prediction model for postoperative mRS scores reached a value of 0.87 ± 0.03 and shows therefore excellent discrimination^53^. This is the highest reported ROC-AUC in ML studies investigating postoperative clinical outcomes in patients with UIAs^15^.

As not every patient had an mRS score of 0 preoperatively, we further introduced the mRS difference into our models, which may be another clinically relevant outcome parameter.

Our MLP model revealed a ROC-AUC of 0.70 ± 0.02. Similarly, a permanent postoperative neurological deficit may be another important parameter that was predicted with a ROC-AUC of 0.71 ± 0.04. Moreover, compared with classical LR, our models revealed a significantly better performance (*p* < 0.0001).

To our knowledge, this is the first study to present ML-based prediction models for functional and clinical outcomes in a large sample of microsurgically treated UIAs using a temporal split.

The pronounced class imbalance in all five outcomes, in conjunction with the relatively small dataset, led to a large variance in the bootstrapped model performance. This also means that the specific train-test split utilized for training and evaluating models can have a huge impact on the final results, as we observed in preliminary experiments with multiple random splits (data not shown). This in turn justifies the nonrandom temporal split.

So far, only a few neurosurgical ML studies were published with an external validation of their models. Good generalisability of external validation is seen in the radiological diagnosis of UIAs^54^ or in the prediction of intracranial aneurysm rupture risk based on multi-omics factors^55^. Fuse et al. published an external validation of their preoperative prediction model for postoperative outcomes after chronic subdural hematoma evacuation and external validation revealed an excellent ROC-AUC of 0.860^56^. However, no external validation of a preoperative prediction model for microsurgically treated UIAs has been published so far^15,16,46^.

In this study, external validation of the best internally validated models shows ROC-AUC values of 0.61, 0.53 and 0.58 respectively for predicting a postoperative mRS > 2, a pre- and postoperative difference in mRS > 1 point and a GOS < 5. This is a poor discrimination of the models in the external validation and therefore the models are not applicable to this tested external dataset from the Department of Neurosurgery at the University Medical Centre Hamburg-Eppendorf.

The prediction models are all based on preoperative parameters. Our SHAP analysis (see Fig. 2a–e) showed that especially the parameters location, symptoms and preoperative mRS have a strong influence on the best-performing models. When these parameters are compared between the internal training and test set and the external validation set (*p*-values in Tables 2 and 3), a significant difference in the underlying population can be seen. The reason for this difference remains unknown and points to the importance of individual centre-specific factors, such as different surgical strategies among different surgeons and different intra- and perioperative setups. As all of the models are trained on the data in a specific setup of a microsurgical high-volume centre, our results clearly show that it has only good predictability for this particular centre. Moreover, our results also clearly demonstrate, that the parameters obtained in the SHAP analysis can be used to check in advance whether a model is not applicable to a certain population. Trustworthiness and transparency as part of a safety net are important for the use of predictive models. Careful validation and adaptation are important when implementing predictive tools in different healthcare settings.

Consistent with the typical distribution of UIAs, this surgical cohort included a large number of middle cerebral artery (MCA) bifurcation aneurysms (n = 309). Aside from Nussbaum et al., it is therefore one of the largest published monocentric registries of microsurgically treated unruptured MCA bifurcation aneurysms^48^. Microsurgical treatment by clipping remains the gold standard for the management of unruptured MCA bifurcation aneurysms, reflecting the clinical importance of our data analysis.

## Limitations

The retrospective nature of the data collection has a limiting effect on the quality of the data registry. All the prediction models were based on a monocentric database over a period of 19 years. Since there were several neurosurgeons with different experiences involved over such a long time, the good results indicate robust predictive models. The diagnostic options and, consequently, the treatment indications for UIAs have changed over the long observation period from 2002 to 2020 and can thus be considered a potential selection bias.

In addition, any prediction model for postoperative outcome parameters based on preoperative parameters underestimates the intraoperative component. The experience or individual decisions of the treating neurosurgeon might have an impact on the outcome. By definition, intraoperative parameters would be possible confounders and thus may not be taken into account in preoperative prediction models.

The chosen outcome parameters were ascertainable and easily comparable. For comprehensive neurocognitive outcome evaluation, a detailed postoperative neurocognitive examination is required.

From a modeling perspective, the feature set was limited to a handful of numerical and categorical variables that could be acquired easily preoperatively. It lacks unstructured information such as imaging data, free-text notes, and medication prescriptions that hold the potential to carry useful information for the prediction tasks considered in this study. Furthermore, one could speculate that ensemble models that combine the decisions of multiple base estimators into one final decision are more accurate than the single-estimator models presented in this study. However, initial experiments with training and tuning ensembles of up to 25 different base estimators led to no or only negligible performance improvements (data not shown) at the cost of considerably more complex, hardly interpretable models.

## Conclusions

In conclusion, the results show excellent and acceptable performances in predicting functional and clinical outcomes after microsurgical therapy of UIAs in the internal validation data set, especially for the main outcome parameters mRS and permanent nND. The application of a temporal train-test split is feasible for this specific question and is unique.

Unfortunately, the excellent models could not be generalized in the external validation data set of an independent neurosurgical department due to major differences between the treated patients and aneurysms in the departments.

The implementation of newly available data and the merging of larger databases to form more broad-based predictive models is imperative.

## Data Availability

The datasets generated and analyzed during the current study are available from the corresponding author on reasonable request.

## Abbreviations

ACA: Anterior cerebral artery
AChA: Anterior choroidal artery
AComA: Anterior communicating artery
ADPKD: Autosomal dominant polycystic kidney disease
AP: Average precision
ASA: American Society of Anesthesiologists
aSAH: Aneurysmal subarachnoid hemorrhage
BMI: Body mass index
CAD: Computer-aided diagnosis
COPD: Chronic obstructive pulmonary disease
Diff.: Difference
DM: Diabetes mellitus
DSA: Digital subtraction angiography
ET: Extremely randomized trees
GAM: Generalized additive model
GOS: Glasgow outcome scale
KNN: k-nearest neighbor classifiers
LDA: Linear discriminant analysis
LR: Logistic regression
MCA: Middle cerebral artery
ML: Machine learning
MLP: Multilayer perceptron
mRS: Modified Rankin Scale
nND: New neurological deficit
NPV: Negative predictive value
PComA: Posterior communicating artery
PPV: Positive predictive value
QDA: Quadratic discriminant analysis
RF: Random forest
ROC-AUC: Area under receiver operating characteristic curve
SAH: Subarachnoid hemorrhage
SHAP: Shapely additive explanations
std.dev.: Standard deviation
SVM: Support vector machines
UIA: Unruptured intracranial aneurysm
XGB: Extreme gradient boosting estimators
